# A Study on the Preparation of Environmentally Friendly High-Performance Natural Rubber Using the Interaction Mechanism of Alkaline Protease and Calcium Ions

**DOI:** 10.3390/polym17040490

**Published:** 2025-02-13

**Authors:** Tuo Dai, Yun Li, Honghai Huang, Li Ding, Jianwei Li, Haoran Geng, Yazhong Song, Tao Zhao, Liguang Zhao, Hongxing Gui

**Affiliations:** 1 Hainan Natural Rubber Technology Innovation Center, Rubber Research Institute, Chinese Academy of Tropical Agricultural Science, Haikou 571101, China; catasdaituo@163.com (T.D.);; 2 Mengla Tianye Rubber Sales Co., Ltd., Xishuangbanna 666100, China

**Keywords:** natural rubber (NR), coagulation, alkaline protease, high-performance, environmentally friendly

## Abstract

Natural rubber (NR) is a material with a wide range of industrial and commercial applications, including agriculture, defense, transportation, and domestic use. However, the mechanical properties of natural rubber treated by traditional acid coagulation are limited, which restricts its application in high-end products. Furthermore, the wastewater generated also causes soil acidification. Consequently, there is a necessity to investigate new coagulation methods to enhance the comprehensive performance of natural rubber and reduce environmental pollution. In this work, a novel method for the preparation of environmentally friendly high-performance natural rubber by alkaline protease/calcium chloride coagulation of natural rubber (AC-NR) is reported. The research demonstrates that the products of protein cleavage by alkaline protease together with calcium ions can greatly enhance the cross-linking between rubber particles, form the network structure of natural rubber well. Furthermore, increasing the pH at the isoelectric point of the discharged wastewater reduces the impact on soil acidification. In comparison with those from conventional acid coagulation of natural rubber (A-NR), the tensile strength of AC-NR was increased by 7.9 MPa, the tear strength was increased by 5.3 kN/m, the final temperature rise was lowered by 6.5 °C, and abrasion performance was improved. This study demonstrates that by utilizing the collaborative impact of alkaline protease and calcium chloride on the rubber molecular chain during the coagulation process of natural rubber, environmentally friendly high-performance natural rubber with excellent mechanical properties and reduced environmental pollution can be prepared without the necessity for chemical modification or cumbersome processes, which is conducive to the green development and high-quality pursuit of NR materials.

## 1. Introduction

Natural rubber (NR) is a natural polymer compound with polyisoprene as the main component, 91–94% of which is rubber hydrocarbon (polyisoprene), and the remainder is non-rubber substances such as proteins, fatty acids, ash, and sugars [1]. As a crucial industrial raw material, natural rubber possesses advantageous properties, including elasticity, plasticity, tensile strength, and resistance to abrasion, making it a widely utilized material in various industrial, agricultural, national defense, transportation, and daily applications [2]. However, due to inherent limitations of the conventional coagulation process and the presence of quality issues in domestic natural rubber, China is compelled to rely on imports for its high-end natural rubber requirements. This dependence poses a major threat to national security and is a ‘bottleneck’ that hinders national development. Furthermore, the wastewater produced by the conventional coagulation process of natural rubber possesses a low pH at the isoelectric point, thereby inducing environmental concerns such as soil acidification [3].

The conventional coagulation process of natural rubber involves acid coagulation, wherein the principle is that H^+^ ions cause the pH at the isoelectric point to decline to a specific critical value. This results in the rubber particles attaining the isoelectric state, thereby eliminating their surface charge and leading to the dissolution of the charge repulsion between the rubber particles. Consequently, the rubber particles adhere to each other and coalesce [4]. However, extensive research has demonstrated that the acid coagulation process results in a significant reduction in the tensile strength of standard rubber. Furthermore, the discharge of rubber wastewater is characterized by high acidity, elevated levels of chemical oxygen consumption (COD), and the presence of other pollutants, thereby causing substantial environmental pollution [5].

The process of natural rubber microbial coagulation involves the utilization of wastewater from rubber gardens and other environments as a medium for the cultivation of microbial strains that possess the capacity to produce acid. This process entails the addition of sugar substances to the original substrate, thereby facilitating continuous decomposition. The cultivation of these microbial strains results in the acidification of the substrate, leading to the production of an acidic liquid that possesses a low isoelectric point. This, in turn, significantly reduces the duration of natural rubber coagulation [6].The duration of natural rubber coagulation has been found to be a crucial factor in the process. The selection of microbial strains that demonstrate effective performance in natural rubber production is less damaging, while also reducing production costs [7]. However, it should be noted that the repeated treatment of fresh latex with alkali (ammonia preservation) and acid (low microbial liquid pH at the isoelectric point) will result in the destruction of the original antioxidant properties of natural rubber, its cross-linking structure, and ageing properties [8].

The coagulation mechanism of natural rubber involves the elimination of the repulsive force between the rubber particles, thereby inducing coagulation. However, there are divergent perspectives on the coagulation mechanism of natural rubber. One perspective attributes the coagulation of natural rubber to the dissociation of salt electrolytes in water, which produces cations. This process leads to the compression of the double layer, resulting in a thinning of the hydration film surrounding the natural rubber particles. The electric potential is reduced to zero, causing the natural rubber particles to lose their repulsive force and agglomerate, leading to coagulation. Alternatively, the presence of two-valent or three-valent metal ions, along with rubber particles derived from fatty acids, has been identified as a crucial factor in the formation of insoluble soaps within the protective layer. This, in turn, has been shown to compromise the stability of the rubber particles, thereby initiating the process of latex coagulation [9]. The pH at the isoelectric point of the natural rubber salt coagulation curve is biased in the weakly acidic region, which reduces the corrosion of instrumentation and environmental pollution caused by acids. However, excessive variable metal ions (e.g., Cu^2+^, Mn^2+^, etc.) accelerate the aging of natural rubber and vulcanized rubber [10], and calcium ions do not affect the aging properties of natural rubber and vulcanized rubber. Studies on the mechanism of action of calcium ions and alkaline protease have shown that alkaline protease has two Ca^2+^ binding sites, and that there is a strong binding force between Ca^2+^ and alkaline protease. Calcium ions and alkaline protease have been shown to greatly increase the activity of alkaline protease after binding calcium ions to alkaline protease [11].

**Figure 1:**
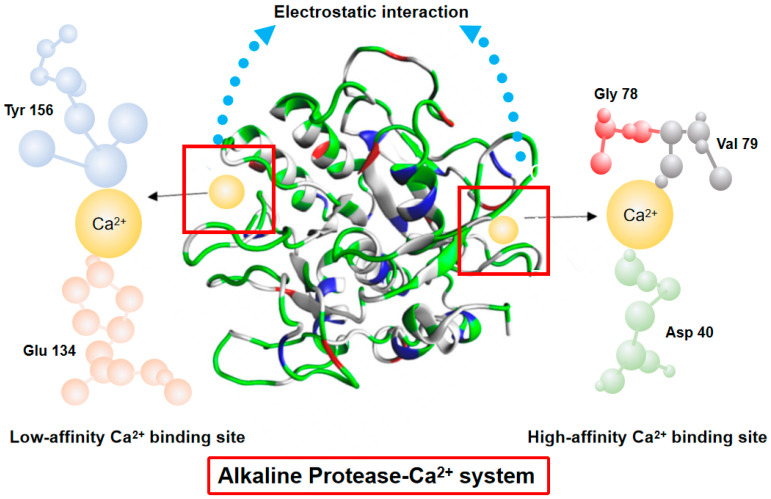
The interaction mechanism between alkaline protease and Ca^2+^.

In this study, a novel technique for natural rubber coagulation using a combination of alkaline protease and calcium ions is proposed, and the mechanism of action of calcium ions to promote alkaline protease activity is demonstrated in Figure 1. The utilization of alkaline protease serves to negate the impact of the low pH at the isoelectric point of acid and microbial coagulation systems on the overall characteristics of natural rubber, as well as the effects of wastewater on soil acidification. The incorporation of calcium ions has been shown to expedite the coagulation process, thereby enhancing the productivity of rubber-processing facilities and optimizing the overall performance of natural rubber. The study also involves a comparative analysis of traditional acid coagulation and microbial coagulation with the novel alkaline protease–salt coagulation technique. This analysis aims to compare the effects of changes in pH at the isoelectric point during coagulation processes on the structure of the rubber network and various performance indicators. The new coagulation technology formed in this project has the potential to enhance the quality of natural rubber, reduce the production cost and wastewater treatment cost of rubber-processing enterprises, and play an important role in promoting the development of high-performance natural rubber industry in China.

## 2. Experimental

### 2.1. Materials

Natural latex from Mengla County Tianye Rubber Sales Ltd., Xishuangbanna, China; Alkaline protease from Nanning Pangbo Bioengineering Ltd., Nanning, China. Acetic acid (98%), Calcium chloride (95%), Zinc oxide (ZnO), Stearic acid (SA), Quebrith, accelerator N-tert-butylbenzothiazole-2-sulfenamide, Concentrated sulfuric acid, Sodium hydroxide, Toluene all from Xilong Reagent Ltd., Shantou, China (Table 1).

### 2.2. Instruments and Equipment

The following items were available from Yuanfeng Inspection Equipment Ltd., Yangzhou, China: an open-type rubber-refining machine, a closed-type rubber-refining machine, a plate-vulcanizing machine, and a Fourier infrared spectroscopy tester. The following items were available from NETZSCH Instruments Ltd., Weimar, Germany: a thermogravimetric analyzer and a differential scanning calorimeter. The following item was available from Shimadzu Ltd., Tokyo, Japan: an electric blast drying box. The following item was available from MonTech Trade Ltd., Buchen, Germany: D-RPA-3000 rubber-processing analyzer.

### 2.3. Preparation of Natural Rubber Samples

**Figure 2:**
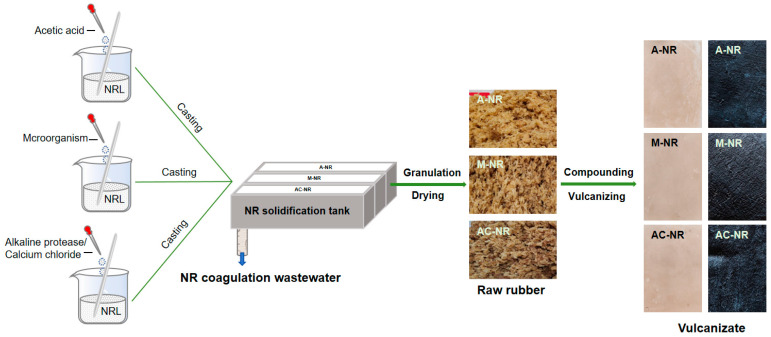
Schematic diagram of natural rubber samples prepared by three coagulation methods.

Natural rubber latex was collected in rubber forests, and the dry rubber content of natural rubber latex (approximately 33–39%) was measured and uniformly diluted to 23%. The initial pH at the isoelectric point of the natural rubber latex was measured and adjusted to neutral, and the natural rubber latex was subsequently coagulated by different coagulation processes. Acetic acid-NR (hereafter referred to as A-NR) was obtained by adding appropriate amounts of acetic acid to the natural rubber latex and mixing uniformly; microorganism-NR (hereafter referred to as M-NR) was obtained by adding appropriate amounts of microbial bacterial liquid to the natural rubber latex. Alkaline protease/calcium chloride-NR (henceforth referred to as AC-NR) was obtained by adding 0.07% alkaline protease and 0.1% calcium chloride to natural rubber latex with homogeneous mixing. The process of natural rubber sample preparation is shown in Figure 2. After the latex had completely solidified, the rubbers were placed in a coagulation tank for 1 day. The wastewater in the three coagulation tanks was extracted to measure the pH at the isoelectric point, and then solidified. The solidified rubber samples were pressed five times, crushed, dried at 110 °C, and extruded to obtain raw rubber blocks, which were mixed. These samples were then subjected to various compounding agents (formulations as shown in Table 2) in the open roll mill, in accordance with the prevailing operating standards [9]. The vulcanization process was then initiated in the vulcanizing machine at 140 °C, yielding vulcanized rubber samples of pure rubber/carbon black formulation.

### 2.4. Characterizations

Gel content: About 0.1 g (noted as m_0_) of natural raw rubber was weighed and soaked for 1 week in tetrahydrofuran as a solvent, then centrifuged by a GL-21M refrigerated centrifuge to get the precipitate. A weighing dish was taken and weighed (m_1_), into which the precipitate was then poured and dried at 100 °C until constant weight, and then the total weight (m_2_) was measured. The gel content (A) was calculated from Equation (1):

(1) $$A=\frac{\left(m_{2}-m_{1}\right)}{m_{0}}\times 100\%$$

Molecular weight size and its distribution: 15 mg of raw rubber was sheared and statically dispersed, then gradually dissolved in a room-temperature tetrahydrofuran (THF) solution, sealed from light, dissolved for 72 h until the sample was completely dissolved, and the sample solution was filtered using LC-100 high-performance gel permeation chromatography (GPC) to determine the relative molecular weight size of the natural rubber samples and its distribution. The test preparation conditions were as follows: polystyrene was the test standard sample, the mobile phase was a THF solution, the concentration of rubber filtrate was 3 mg/mL, and the flow rate was 1 mL/min. The test preparation conditions were as follows: polystyrene was used as the test standard, the mobile phase was a THF solution, the concentration of rubber filtrate was 3 mg/mL, the flow rate was 1 mL/min, the temperature of the test sample was stabilized at 30 °C, and the injection volume was 10 μL.

Vulcanization characteristics: 5 g of rubber mixing samples were taken to determine the vulcanization characteristics of natural rubber using a CZ-3001D rotorless rheometer, 143 °C × 60 min.

Infrared spectroscopy: Preparation of thin samples of natural rubber, a Nicolet iS5 Fourier infrared spectrometer was used to perform attenuated total reflectance (ATR) infrared spectroscopy scanning on thin samples. The necessary conditions for the test were: wave number scanning range of 600–4000 cm^−1^, spectral image resolution of 4 cm^−1^, and the number of scanning completion of 16.

Physicochemical indicators: Mooney viscosity test was performed according to the GB/T1232.1-2000 standard by taking homogenized raw rubber samples of about 8 g, sourced from Abundant Testing Equipment Co., Ltd. (Delhi, India) and produced by a YF-8005 type Mooney viscosity meter. The initial value of plasticity (P_0_) was tested using a KS-II rapid plasticity meter at 100 °C, preheated for 1min and tested for 4 min. P_30_ was tested using an aging test chamber for aging the samples (140 °C, 30 min) and tested using a KS-II rapid plasticity meter. The plasticity retention rate (PRI) was calculated according to Equation (2):

(2) $$PRI=\frac{P_{30}}{P_{0}}\times 100\%$$

**Figure 3:**
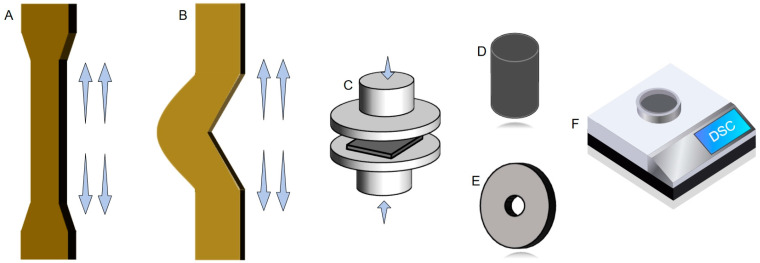
(**A**) Schematic of tensile strength test. (**B**) Schematic of tear strength test. (**C**) Schematic of RPA test of dynamic mechanical properties. (**D**) Schematic of DIN abrasion sample. (**E**) Schematic of Akron abrasion sample. (**F**) Schematic of the DSC instrument.

Physical and mechanical properties: Natural rubber samples were vulcanised and prepared in the shape of a dumbbell and hardness and thickness were measured to test the tensile strength of natural rubber. Natural rubber samples were prepared in right-angle shape after vulcanisation and the thickness was measured to test the tear strength of natural rubber. Tensile strength and tear strength were measured on a PT-307 universal material tensile machine produced by Puzet Testing Equipment Ltd., Quanzhou, China. with tensile speed of (500 ± 50) mm/min (Figure 3A,B).

RPA test dynamic mechanical properties: D-RPA-3000 rubber-processing analyzer test was used to evaluate the rheological properties of the natural raw rubber samples. Regarding the Payne effect (ΔG′), the rubber was mixed at 100 °C, with 0.5 Hz strain scanning 0.28–1000%, and then warmed up to 143 °C to complete the vulcanization (time 1.2 × t_90_min), and finally the Payne effect was calculated according to the following formula: ΔG′ = G′0.28–100%. Finally, the temperature was lowered to 100 °C to complete the strain scan test on the vulcanized rubber (range 0.28–100%), and the Payne effect was calculated according to the following formula: ΔG′ = G′0.56–G′100% (Figure 3C).

Compression heat generation: The height, diameter, and hardness of the prepared cylindrical vulcanized rubber samples were measured, and a compression fatigue temperature rise testing machine RH-2000N was used to carry out the heat generation test of the samples. The sample was preheated for 30 min, the temperature was generally constant at 55 ± 1 °C, the stroke was 4.45 mm, the load was 1.0 MPa, the test frequency was set at 20 Hz. The sample was preheated for 30 min and then tested for 25 min, and the final temperature rise of the sample was recorded.

DIN abrasion: Cylindrical samples of natural rubber vulcanised with carbon black were prepared first (Figure 3D). The test method involved the utilization of a DIN abrasion tester, wherein the specimen was pressed against a rotating drum coated with sandpaper, with a contact pressure of 10 N. The drum was set to rotate at a speed of 40 r/min, and the specimen was moved horizontally over the drum. The wear volume of the rubber specimen was then measured after a stroke of 40 m. It is imperative to note that the test was conducted in a circular motion, with the specimen being moved horizontally over the drum.

Akron abrasion: A long sample of carbon black vulcanised natural rubber was first prepared, the sample was fixed on a rubber wheel axle and the motor was started (Figure 3E). After pre-grinding for 15 min (500 revolutions), the sample was removed, the crumbs were brushed, weighed, and recorded as m_1_. The test was carried out with a pre-milled sample. After driving 1.61 km (3418 revolutions) [12], the motor was turned off, the sample was removed, brushed with the crumbs, and weighed within 1 h, recorded as m_2_. The density ρ of the sample was measured using a rubber gravimeter. The wear volume (V) of the specimen was calculated as follows:

(3) $$V=\frac{m_{1}-m_{2}}{\rho }$$

Thermogravimetry analysis: The thermal degradation of raw rubber samples was studied in depth using a thermogravimetric analyzer (TGA 101). First, 5–10 mg of raw rubber samples were cut into pieces and placed in an alumina crucible in the TG sample chamber. The experimental conditions consisted of a nitrogen atmosphere, with a constant nitrogen flow rate of 20 mL/min, and a temperature increase rate that varied at 10 °C per minute. The temperature interval ranged from 30 to 550 °C.

Differential scanning calorimetry: The glass transition temperature (Tg) of the raw rubber samples was tested using a JB-DSC-500 differential scanning calorimetry (DSC) analyzer, and 5–10 mg samples were cut into pieces and put into an aluminum crucible, placed in the sample chamber of DSC (Figure 3F), and the process of going from the low temperature of −80 °C to the high temperature of 100 °C was repeated twice to eliminate the thermal effect. The test conditions were as follows: nitrogen atmosphere, temperature increases and decreases at a rate of 10 °C per minute, and temperature range of −80 to 100 °C, and nitrogen and liquid nitrogen flow rate of 20 mL/min.

## 3. Results and Discussion

### 3.1. Changes in pH at the Isoelectric Point of Natural Rubber During Coagulation with Different Coagulation Methods

Natural rubber latex, composed of rubber particles of varying sizes, exhibited polydispersity, thereby resulting in inconsistent stability amongst the particles. The most unstable particles, approaching the isoelectric point of cohesion, initially exhibited a tendency to congregate in proximity to the isoelectric point, gradually disseminating to all rubber particles over time. In the event that the isoelectric point of latex is not fully attained, but instead only approaches the pH level, the coagulation process of natural rubber is deemed incomplete. This phenomenon is referred to as “natural rubber seepage white water”. Consequently, the coagulation process is not instantaneous, but rather undergoes a series of changes during its progression [9]. The study of the pH at the isoelectric point change in this process is of great significance to investigate the changes in the internal composition and network structure of natural rubber in different coagulation methods, and the effect of wastewater on the degree of soil acidification.

**Figure 4:**
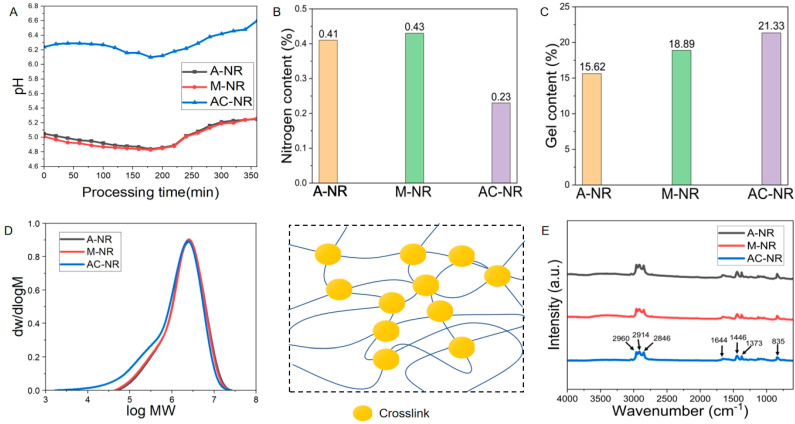
(**A**) Trends in pH at the isoelectric point during the coagulation of NR. (**B**) Nitrogen content of NR. (**C**) Gel content of NR. (**D**) Molecular weight distribution of NR. (**E**) Infrared spectra of NR.

Figure 4A illustrates the alterations in pH at the isoelectric point of A-NR, M-NR, and AC-NR throughout the coagulation process. The initial and overall pH at the isoelectric point of A-NR and M-NR were lower than that of AC-NR due to the lower pH at the isoelectric point of the added coagulant. The overall trend of pH at the isoelectric point of the three types of coagulation modes (i.e., A-NR, M-NR, and AC-NR) was shown to be the same, with a decrease first and then an increase. The time period from 0 to 60 min was shown to be the time period for alkaline protease of bacillus a class of serine protease. The hydrolysis reaction for a protein by serine proteases is carried out by combining active sites with serine proteases. The hydrolysis reaction for a protein by serine proteases involves the combination of active sites with substrates, resulting in the production of a histidine containing the imidazole group and a serine residue with a β-OH as a functional group and other basic amino acids [11]. These amino acids have a pH > 7 at the isoelectric point [13], leading to a modest increase in the pH at the isoelectric point of AC-NR. A-NR and M-NR are devoid of alkaline protease, the enzyme responsible for the breakdown of proteins to produce alkaline amino acids. Instead, these microorganisms are capable of producing acidic substances through a different mechanism. Consequently, the pH at the isoelectric point of A-NR and M-NR was decreasing since the onset of the reaction. Furthermore, a substantial number of exotic microorganisms are present in M-NR, which serve to augment the “acidification” reaction of the original substrate [14]. This further exacerbates the decline in pH at the isoelectric point.

In the 60–180 min time period, the coagulation of natural rubber was observed. The process of microbial reproduction in natural rubber leads to the decomposition of its organic matter, resulting in the production of a substantial quantity of acidic substances, namely A-NR, M-NR, and AC-NR. This process concurrently results in a decrease in the pH at the isoelectric point of the material. After 180 min, the three coagulation methods of NR were found to be essentially solidified. The coagulation of natural rubber is characterized by the mechanism of “agglomerate maturation”. At this stage, H^+^ participates in the construction of the network between natural rubber hydrocarbons and is consumed. This process serves as a conduit, connecting the rubber particles [9]. As H^+^ is utilized and reduced within the natural rubber coagulation system, the pH at the isoelectric point in the residual liquid is observed to undergo an increase. Consequently, A-NR, M-NR, and AC-NR attained a higher final pH at the isoelectric point compared to that at the initial stage of coagulation. The alkaline protease enzyme dissolves the bound proteins on the surface of the rubber particles, and the decomposition products of the proteins and the dimethylallyl groups on the end groups of the molecular chains are connected by van der Waals forces, hydrogen bonding, or ionic bonding. This process leads to the formation of omega-terminal groups and the establishment of a cross-linking network structure [15,16]. Calcium ions act as ionic bridges between rubber particles and H^+^ ions during the coagulation reaction [9]. The interplay between these two effects results in the entanglements of additional rubber molecular chains, thereby enhancing the network structure of natural rubber and improving its quality.

The prolongation of the “agglomerate ripening” time of natural rubber results in a more compact internal network structure, with continuous consumption of H^+^ and a pH at the isoelectric point of the liquid phase that is AC-NR > A-NR ≈ M-NR. The wastewater from the coagulation of AC-NR is weakly acidic, which has less impact on soil acidification and rubber tree growth than the strongly acidic wastewater from A-NR and M-NR [17]. In conclusion, it can be posited that AC-NR possesses superior quality and also mitigates the effect of wastewater from natural rubber processing on soil acidification.

### 3.2. Effect of Different Coagulation Methods on Gel Content of Natural Rubber

Gel content is employed to denote the macromolecules present within natural rubber that are insoluble in organic solvents, such as toluene. These macromolecules have the capacity to respond to the extent of protein and rubber molecule cross-linking with each other, thereby resulting in the formation of a network structure [18]. As demonstrated in Figure 4B,C, which depicts the protein content and gel content respectively, under three distinct coagulation modes, it is evident that AC-NR exhibited reduced protein content, accompanied by augmented gel content. This phenomenon can be attributed to the process of alkaline protease digestion of proteins, which results in the production of amino acids. The opening of the epoxy group on the rubber molecular chain, consequent to this process, serves to promote the cross-linking of molecular chains; at the same time, calcium ions play a role in the rubber particles between the “bonding bridge”. These two factors influence the micro-cross-linking network structure in the AC-NR coagulation system more than in the A-NR and M-NR coagulation systems. The nature of the A-NR and M-NR coagulation systems is such that the addition of strong acid reagents leads to the isoelectric point of natural rubber within a short period of time, causing a strong change in the stability of the coagulation and also damaging the hydrogen bond formation of the rubber and the coagulation of the rubber. In addition, it has been demonstrated that the process of A-NR and M-NR coagulation results in the destruction of the pivot point of hydrogen bond formation [19]. This phenomenon resulted in a reduced degree of cross-linking of the terminal groups of the rubber molecular chains in A-NR and M-NR, and the gel content of both was lower than that of AC-NR.

### 3.3. Effects of Different Coagulation Methods on Molecular Weight Size and Distribution of Natural Rubber

As illustrated in Table 3, the number average molecular weight (Mn), the weight average molecular weight (Mw), and the distribution coefficients of A-NR, M-NR, and AC-NR are reported. The molecular weight size and its distribution represent the basic structural parameters of natural rubber polymers polymerized from repeated rubber hydrocarbon units. The number of macromolecular structures contained in natural rubber has an impact on the molecular weight size [20], so the most appropriate method for determining the molecular weight size is to use Mw (weight average molecular weight). It is evident that the number average molecular weight (Mn) of the three coagulation modes of natural rubber did not vary significantly, with AC-NR exhibiting the largest weight average molecular weight of 24.15 × 10^5^ daltons. The molecular weight distribution in Figure 4D reveals a substantially larger area for AC-NR in comparison to that of A-NR and M-NR. The results of molecular weight size and gel content also correspond to each other, which can be attributed to the presence of rubber molecules with aldehyde-containing groups. These undergo condensation reactions with other groups to produce cross-linking between rubber molecules [21], and these condensation reactions increase the molecular weight of rubber and gel content [22]. The low pH at the isoelectric point of A-NR and M-NR has been shown to affect the formation of physical cross-linking bonds in natural rubber, leading to an increase in the proportion of low molecular weight compounds and a decrease in the molecular weight of natural rubber.

The molecular weight distribution curves of the same fresh latex raw materials were found to be largely similar, a bimodal distribution with one shoulder and one peak in both the low and high molecular weight regions. AC-NR was co-treated with alkaline protease and calcium ions, which resulted in an augmentation of the “phospholipid-α-terminal group” network structure, accompanied by an enhancement in the heavy average molecular weight of natural rubber (Mw) and a broadening of the distribution [23].

### 3.4. Influence of Different Coagulation Methods on Vulcanization Characteristics of Natural Rubber

As illustrated in Table 4, the torque values and vulcanization rates of A-NR, M-NR, and AC-NR during vulcanization are presented. It is evident that the difference between the highest torque (M_H_) and the lowest torque (M_L_) of AC-NR was the largest at 6.88 dN·m. The magnitude of M_H_-M_L_ has been shown to reflect the number of cross-linking bonds formed in natural rubber during vulcanization [24], indicating that alkaline protease and calcium ions facilitate the formation of more cross-linking bonds and greater cross-linking density in natural rubber. Scorch time is defined as the time required for torque to reach 10%, indicating the duration of the heat accumulation effect of natural rubber during the mixing of the filler. The remaining scorch time is the time that the rubber can flow before setting, and the scorch time (t_10_) and the optimum curing time (t_90_) of the AC-NR were shorter than those of the A-NR and the M-NR. This was due to the fact that the alkaline protease decomposed proteins and produced a large number of peptide bonds, and the amide group of the peptide bonds formed a ligand complex with the zinc ions in zinc oxide, increasing the solubility of zinc oxide in the vulcanization reaction matrix, thus playing a role in promoting vulcanization. Within the vulcanization reaction system, an increase in the concentration of zinc oxide is observed, and the primary formation between the promoter and sulfur is a brief cross-linking bond, which increases the active site of the cross-linking reaction [25]. It can be discerned that alkaline protease and calcium ions have the capacity to regulate the number of cross-linking bonds and scorch time of natural rubber during the vulcanization process, thereby affecting the comprehensive performance of natural rubber.

### 3.5. Comparison of Infrared Spectra of Natural Rubber Under Different Coagulation Methods

As illustrated in Figure 4E, the infrared spectra of AC-NR, M-NR, and A-NR exhibited characteristic peaks that demonstrated comparable trends, suggesting that the incorporation of alkaline protease and calcium ions into natural rubber did not induce substantial alterations to the main chain or branched structures. This observation signifies that natural rubber maintains its inherent benefits over synthetic rubber. There, 2960 cm^−1^ corresponded to the antisymmetric stretching vibration peak of -CH_3_, 2960 cm^−1^ corresponded to the antisymmetric stretching vibration peak of -CH_3_, 2914 cm^−1^ corresponded to the antisymmetric stretching vibration peak of -CH_2_-, and 2846 cm^−1^ corresponded to the symmetric stretching vibration peak of -CH_2_-. The amide I band absorption peak at 1644 cm^−1^ [26] is indicative of protein presence in the latex, with the area under the amide I vibration absorption band corresponding to the protein content. This suggests that the protein content of AC-NR was low. The peaks at 1446 cm^−1^ and 1373 cm^−1^ corresponded to methyl δC-H symmetric bending vibrations, while the peak at 835 cm^−1^ was attributed to C-H deformation vibrations external to the plane of the natural rubber cis-structure. It is evident that natural rubber maintained its distinct advantageous properties and enhanced its overall performance when subjected to the addition of alkaline protease and calcium ions.

### 3.6. Effects of Different Coagulation Methods on Physical and Chemical Indexes of Natural Rubber

**Figure 5:**
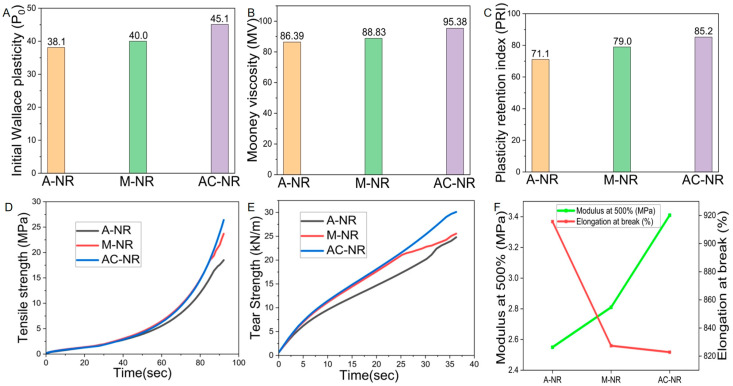
(**A**) Initial plastic value P_0_ of NR. (**B**) Mooney viscosity of NR. (**C**) Plasticity retention index (PRI) of NR. (**D**) Tensile strength of NR. (**E**) Tear strength of NR. (**F**) Comparison of trends in elongation at break and 500% modulus of NR.

As demonstrated in Figure 5A,B, the initial plasticity value P_0_ and Mooney viscosity of AC-NR were greater than those of A-NR and M-NR. The initial plasticity value P_0_ is indicative of the plasticity of natural rubber, which has a larger correlation with gel content, molecular weight, and mechanical properties of natural rubber [27], which suggests that AC-NR samples have more network structures formed by intertwining the molecular chains of the rubber molecules in the coagulation process, but it will slightly reduce its plasticity. Mooney viscosity is a significant indicator of the performance of natural rubber processing, primarily employed to reflect the processing difficulty of natural rubber. An elevated Mooney viscosity value indicates poor natural rubber plasticity, which in turn affects the subsequent calendering and extrusion operations. This can result in difficulties in achieving uniformity in the rubber mixed filler, and in the subsequent extrusion process. Conversely, if the Mooney viscosity value is too low, natural rubber can adhere to the rollers, which can have a detrimental effect on the calendering and extrusion operations [28]. As AC-NR contains alkaline protease, it is reasonable to hypothesize that a greater number of binding proteins will be consumed in the rubber molecular chain. Furthermore, it is predicted that an increased number of proteins will be forced to undergo cross-linking to build a network structure, which may impede the flow between rubber molecules. The hypothesis is supported by the higher Mooney viscosity of AC-NR. The plasticity retention index (PRI) in Figure 5C represents the oxygen ageing resistance of natural rubber, which is positively related to the oxygen ageing resistance of natural rubber [29]. Protein is a natural antioxidant [30], and AC-NR had the lowest protein content but the highest plasticity retention PRI, which may have been due to the addition of alkaline protease and calcium ions to AC-NR. The variable metal particles in natural rubber cause electron transfer when heated, which promotes the generation of free radicals and thus the decomposition of rubber hydrocarbons [31]. Amino acids, produced by the breakdown of proteins by alkaline proteases, have been shown to bind readily to metal particles, reducing the abundance of metal ions and thereby inhibiting the free radical chain reaction, thus slowing down the aging of rubber [32].

### 3.7. Influence of Different Coagulation Methods on Physical and Mechanical Properties of Natural Rubber

The physical and mechanical properties of natural rubber are significant metrics for evaluating its quality, particularly in relation to its application in various rubber product areas. These properties must be met to ensure compliance with production and utilization requirements, both in the formulation of products and the design of processes. As demonstrated in Figure 5D, which illustrates the tensile strength of the samples, AC-NR exhibited higher tensile strength in comparison to that of A-NR and M-NR. This indicates that AC-NR possesses a superior ability to withstand maximum tensile stress during the fracture process when compared to that of A-NR and M-NR. Furthermore, this finding suggests that the destructive force of AC-NR is greater than that of AC-NR under the action of a certain external force [33]. This suggests that AC-NR has the capacity to enhance polar substituents and crystallinity within the rubber molecular chain in the presence of alkaline protease and metal ions, thereby augmenting the intermolecular forces between the rubber molecules [34]. This phenomenon is absent in the acid-solidified A-NR system. The Figure 5E represents the tear strength, and the findings demonstrate that AC-NR exhibited superior tear strength in comparison to that of A-NR and M-NR. This observation is consistent with the comparative results of MH-ML. The reason for this is that there is a strong correlation between tear strength and cross-linking density. In a specific cross-linking density range, the tear strength increases with the increase of cross-linking density [35]. The results demonstrate that AC-NR exhibited the capacity to resist continuous rupture at the crack under unit thickness.

**Figure 6:**
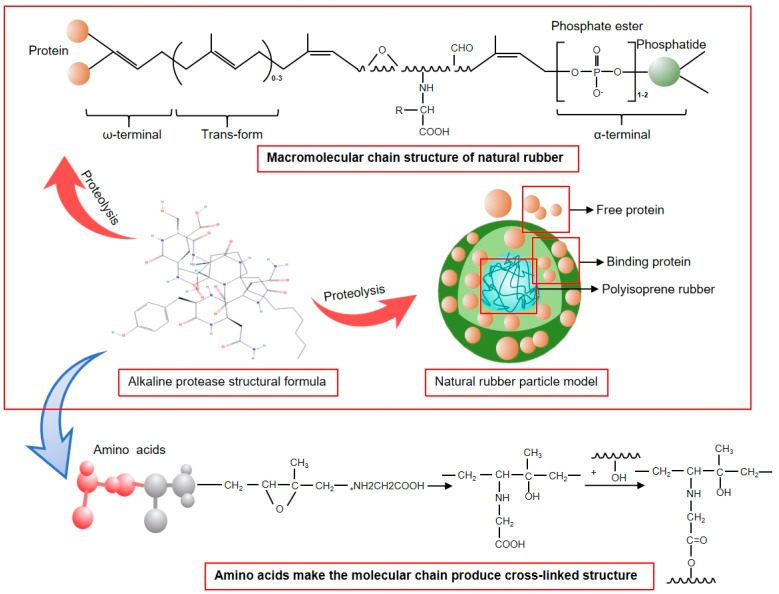
Alkaline protease enhances overall performance of NR.

As demonstrated in Figure 5F, the modulus at 500% and the elongation at break of A-NR, M-NR, and AC-NR exhibited a close correlation with the network-like structure of rubber hydrocarbon linear polymers, which were formed by cross-linking following the vulcanization of natural rubber [36]. Furthermore, an inverse proportional relationship was observed between these two parameters. The modulus at 500% of AC-NR and M-NR were 3.41 MPa and 3.21 MPa, respectively, which were higher than that of A-NR [37]. Figure 6 illustrates the potential mechanisms through which alkaline proteases enhance the overall properties of natural rubber. With the increase of cross-link density, constant tensile stress increased, which indicates that AC-NR vulcanized rubber built more network structures to resist external deformation, but there was a partial loss of resilience and increased permanent deformation of AC-NR vulcanized rubber, and the elongation at break was at the normal level, although slightly lower.

### 3.8. Effects of Different Coagulation Methods on the Processing Properties of Natural Rubber

**Figure 7:**
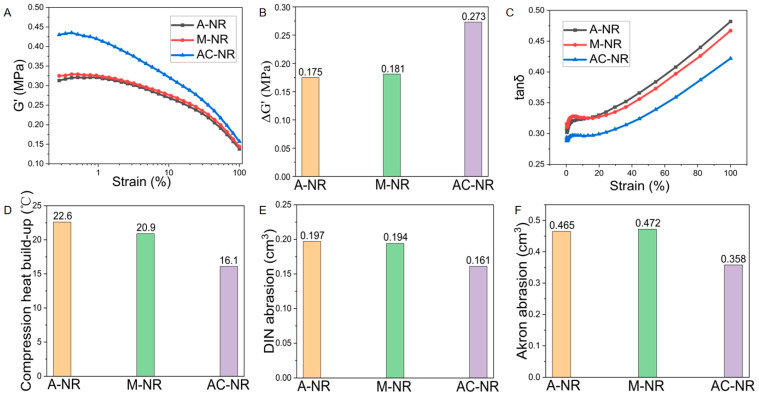
(**A**) Elastic modulus G′ of NR. (**B**) Payne effect of NR. (**C**) Dielectric loss factor tanδ of NR. (**D**) Final temperature rise of NR. (**E**) DIN abrasion of NR. (**F**) Akron abrasion of NR.

As natural rubber is subject to dynamic influence during processing and use, the testing of dynamic mechanical properties can provide an objective assessment of the comprehensive performance of natural rubber, thereby characterizing the viscoelastic trend throughout the process before and after vulcanization of the rubber material [38]. The trends of energy storage modulus G′ for A-NR, M-NR, and AC-NR are represented in Figure 7A, and it is evident that the trends were essentially similar within the same strain angle range. AC-NR exhibited the highest energy storage modulus G′, indicating its greater gel content, extensive molecular chain branching and cross-linking, and pronounced rigidity. As demonstrated in Figure 7B, AC-NR displayed a pronounced Payne effect (ΔG′), which was particularly evident under cyclic loading conditions with a large strain amplitude, as evidenced by the dependence of the viscoelastic energy storage modulus on the amplitude of the applied strain. When the strain amplitude was greater than 0.1%, the increased friction generated endogenous heat due to the intermolecular movement of natural rubber inside the fracture. The molecular chain of the rubber, composed of a cross-linked network, was easily destroyed, resulting in a slow decrease in the AC-NR storage modulus with an increasing amplitude. At a strain amplitude of 20% or greater, the storage modulus of natural rubber decreased rapidly and tended to a lower limit value, in the region where the decrease in the storage modulus G′ was accompanied by an increase in the loss modulus G″ [39], and this tendency was reflected in the trend of the value of the dielectric loss factor tanδ (Figure 7C). The dielectric loss factor tanδ of AC-NR was minimal, indicating that the alkaline protease enzyme caused cross-linking between the rubber molecular chains to form a network of entanglements. This has been shown to increase the rolling resistance of natural rubber during processing, resulting in a decrease in the processing performance of the material.

### 3.9. Influence of Different Coagulation Methods on Thermal Performance of Natural Rubber

The final temperature rise is defined as the phenomenon of delayed dissipation of heat that occurs when the molecular chains of rubber rub against each other under high-frequency loaded compression vibration of a vulcanized rubber cylinder sample [40]. Vulcanized rubber samples generate and accumulate heat and radiate it throughout the compression resilience test, and the lower the final temperature rise, the better the heat generation performance [41]. As demonstrated in Figure 7D, the final temperature rises of A-NR, M-NR, and AC-NR were 22.6 °C, 20.9 °C, and 16.1 °C, respectively, with AC-NR exhibiting the optimal thermogenic performance. This phenomenon can be attributed to the reduced protein content of AC-NR, where the presence of rubber particles with low protein content facilitates the reduction of deformation through particle rotation, thereby diminishing endogenous heat generation [42]. The findings demonstrate the efficacy of the alkaline protease–salt coagulation system in mitigating the final temperature rise of natural rubber, thereby enhancing its heat generation performance to a certain extent.

### 3.10. Influence of Different Coagulation Methods on the Abrasion Performance of Natural Rubber

DIN abrasion tests the static loss of natural rubber, and Akron abrasion tests the dynamic loss of natural rubber (see Figure 7E,F). These figures show that AC-NR had the best abrasion performance, with the smallest DIN abrasion loss volume and the smallest Akron abrasion loss volume of 0.161 cm^3^ and 0.358 cm^3^, respectively. This finding suggests that alkaline protease and calcium ions could enhance the structure of natural rubber molecular chain entanglement and cross-linking network construction, thereby improving resistance to physical friction damage. Furthermore, the final temperature rise of AC-NR was also the lowest, indicating that the improvement of natural rubber’s thermogenic properties could reduce the damage caused by frictional heat to the network structure. DIN abrasion is a method of evaluating the abrasion resistance of natural rubber used in the resistance of natural rubber to abrasion in the manufacturing of softer rubber flooring and hose compounds, while Akron abrasion is utilized to assess the abrasion resistance of harder shoe soles and tire compounds. These findings suggest that AC-NR has a wide range of applications in the field of both soft and hard rubber products, thereby underscoring its versatility and significance in various industrial contexts.

### 3.11. Thermogravimetric Change Analysis of Natural Rubber Under Different Coagulation Methods

**Figure 8:**
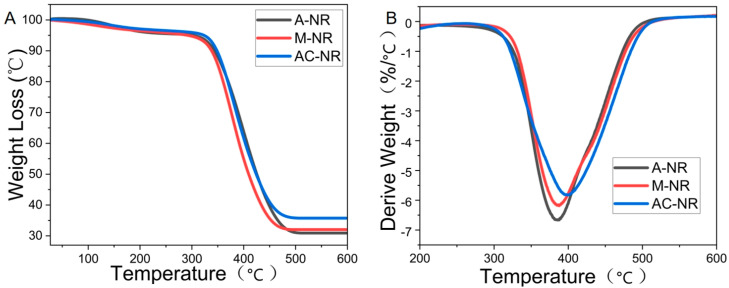
(**A**) Thermogravimetric curve of NR. (**B**) DTG curve of NR.

The ability of natural rubber structures to withstand impacts at high temperatures, and the thermal stability relationship between mass and temperature were evaluated by thermogravimetric analysis [43]. As demonstrated in Figure 8A, natural rubber undergwent a thermal degradation reaction, which is a smooth curve under a nitrogen atmosphere, and is a one-step reaction with one peak. The thermogravimetric (TG) curves were processed using software employing the double tangent method to obtain three heat loss characteristic temperatures: T_O_ (onset of degradation temperature), T_P_ (maximum degradation rate temperature), and T_f_ (termination of degradation temperature). The natural rubber temperature curves were calculated for percentage of mass consumption at T_P_ and T_f_ to the two degradation rates, degradation rate at peak (Cₚ) and final degradation rate (Cf), were then obtained. As presented in Table 5, the highest T_O_, T_P_, and T_f_ were found for AC-NR, with 329 °C, 401 °C, and 510 °C, respectively, which indicates the better thermal stability of AC-NR. The residual mass of AC-NR was the largest among the three, which also verifies that AC-NR had good thermal stability. The temperature at which the maximum rate of mass change occurred (i.e., the peak of the DTG curve in Figure 8B) is indicative of the thermal stability of AC-NR, with the peak temperature of AC-NR occurring on the rightmost side of the curve. A comparison of the degradation rates at T_P_ and T_f_ temperatures reveals that the characteristic temperature thermal degradation rates C_P_ and C_f_ of AC-NR were the lowest, indicating that the addition of alkaline protease and calcium ions could improve the thermal stability of natural rubber and better fight against its own decomposition at high temperatures.

### 3.12. Influence of Different Coagulation Methods on Glass Transition Temperature of Natural Rubber

When natural rubber is at the glass transition temperature (Tg), the specific heat capacity, pressure and expansion modulus coefficients, and viscosity all undergo a sudden change [44]. As shown in Table 6, the glass transition temperature (Tg) of AC-NR was −59.62 °C, which was the highest among the three. In the context of biomolecular structure, at the point of complete glass transition, the molecular chain of natural rubber is unable to move freely, but one segment can begin to move rapidly, due to the smooth nature of the rubber molecular chain segments, which exhibit a high-speed rebound. As the temperature continues to rise, the interconnected molecular chains of natural rubber are prompted to move rapidly in the direction of non-rebounding, resulting in the production of viscous fluidity on the surface [45]. The temperature change of AC-NR is distinct from that of A-NR and M-NR due to the decomposition of proteins by alkaline protease and the reaction of other rubber molecules with each other, thereby enhancing the cross-linking reaction between the rubber, and as the force between the molecular chains of proteins gradually increases, so too does the energy required for movement. Consequently, the glass transition temperature Tg of natural rubber is slightly accelerated, moving slightly towards higher temperatures.

## 4. Conclusions

In this study, natural rubber samples with three different coagulation systems (i.e., acid, microbial, and alkaline protease/calcium chloride) were prepared in order to compare the differences in the comprehensive performance indexes of natural rubber under the new coagulation system and traditional coagulation systems. The investigation encompassed a comprehensive set of parameters, including the pH at the isoelectric point change during the coagulation of fresh rubber latex; protein content, gel content, molecular weight size and distribution, cross-linking density and scorch time, physicochemical indexes, mechanical properties, processing properties, thermal properties, abrasion properties, and other performance indexes of raw rubber. The results demonstrated that the utilization of alkaline protease/calcium chloride coagulation of natural rubber, in comparison with conventional acid coagulation, was capable of reducing the impact on soil acidification, enhancing molecular chain entanglement, gel content, and molecular weight of natural rubber, increasing tensile strength by 7.9 MPa, increasing tear strength by 5.3 kN/m, reducing the final temperature rise by 6.5 °C, and improving abrasion performance. In summary, the experimental results provide theoretical support for the preparation of environmentally friendly, high-performance natural rubber. The addition of alkaline protease and calcium chloride to the coagulation process of natural rubber has been shown to yield superior overall performance, while concomitantly reducing production costs and wastewater treatment expenses for rubber-processing enterprises. Consequently, this approach can be widely applied in the field of natural rubber primary processing.

## Figures and Tables

**Table 1:** Types and sources of main raw materials.

Materials	Specification	Manufacture Factory
Nature rubber	LR	Mengla County Tianye Rubber Sales Ltd., Xishuangbanna, China
Alkaline protease	AR	Nanning Pangbo Bioengineering Ltd., Nanning, China
Calcium chloride	AR	Xilong Reagent Ltd., Shantou, China
Acetic acid	AR	Xilong Reagent Ltd., Shantou, China
Zinc oxide (ZnO)	AR	Xilong Reagent Ltd., Shantou, China
Stearic acid (SA)	AR	Xilong Reagent Ltd., Shantou, China
Sulfur(S)	AR	Xilong Reagent Ltd., Shantou, China
TBBS	AR	Xilong Reagent Ltd., Shantou, China
Accelerator M	AR	Xilong Reagent Ltd., Shantou, China
Carbon black	AR	Xilong Reagent Ltd., Shantou, China

**Table 2:** Vulcanization formulas of NR.

Samples	NR	ZnO	Sulfur	Stearic Acid	Accelerator M	TBBS	Carbon Black
Formula of pure rubber compound, g	100	6	3.5	0.5	0.5	—	—
Formula of carbon black rubber compound, g	100	5	2.25	2	—	0.7	35

**Table 3:** Molecular weight size and distribution of NR.

Samples	Number Average Molecular Weights/Mn	Weight Average Molecular Weight/Mw	Molecular Weight Distribution Width Coefficient/Mw/Mn
A-NR	3.01 × 10^5^	11.05 × 10^5^	3.67
M-NR	3.45 × 10^5^	13.35 × 10^5^	3.87
AC-NR	3.51 × 10^5^	24.15 × 10^5^	6.88

**Table 4:** Vulcanization characteristics of NR.

Samples	M_H_/dN·m	M_L_/dN·m	M_H_-M_L_/dN·m	t_10_/min	t_90_/min
A-NR	0.50	6.21	5.71	2:01	17:57
M-NR	0.52	6.58	6.06	1:56	18:25
AC-NR	0.79	7.08	6.29	1:31	12:25

**Table 5:** Characteristic temperature and degradation rate of NR.

Samples	T_O_/°C	T_P_/°C	T_f_/°C	C_P_/%	C_f_/%
A-NR	344	365	381	47	80
M-NR	347	369	390	45	79
AC-NR	350	373	396	40	76

**Table 6:** Glass transition temperature of NR.

Samples	Glass Transition Temperature/°C
A-NR	−60.58
M-NR	−60.15
AC-NR	−59.77

## Data Availability

The data presented in this study are available on request from the corresponding author.
